# Supplementary material for: MEG-GPT: A transformer-based foundation model for magnetoencephalography data
Source: Imaging Neurosci (Camb). 2026 Jul 24;4:IMAG.a.1301. doi: 10.1162/IMAG.a.1301 (PMC13403652; doi:10.1162/IMAG.a.1301)
Supplement: Supplementary Material [file IMAG.a.1301_supp.pdf]

Supplementary Information (SI)

A Extra details regarding the tokeniser

Percentage of variance explained

The percentage of variance explained (PVE) is defined by

$$PVE = 100 \times \left(1 - \frac{\sum_{t=1}^{T}\sum_{c=1}^{C}(x_t^{(c)} - \tilde{x}_t^{(c)})^2}{\sum_{t=1}^{T}\sum_{c=1}^{C}(x_t^{(c)})^2}\right)\%, \tag{A.1}$$

where x is the real data, $\tilde{x}$ is the reconstructed data, T is the number of time points and C is the number of parcels.

Hyper-parameters and training curve

Here we present the hyper-parameters of the tokeniser in Table A.1 and the training curve in Figure A.1.

**Model parameters**			
Number of tokens K	128	Token width d_{token}	10
GRU number of units	128	GRU sequence length	200
**Training parameters**			
Batch size	32	Number of epochs	10
Learning rate	1e-5		

Table A.1: Hyper-parameters for the tokeniser.

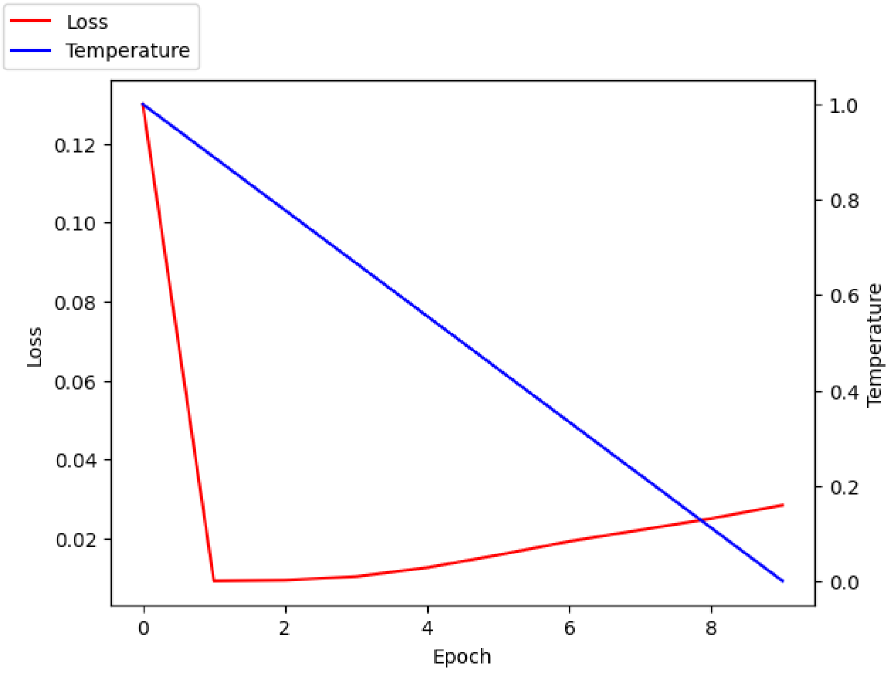

Figure A.1: **Training curve of the tokeniser**. The training loss is plotted in red against the epochs and the temperature during annealing is plotted in blue.

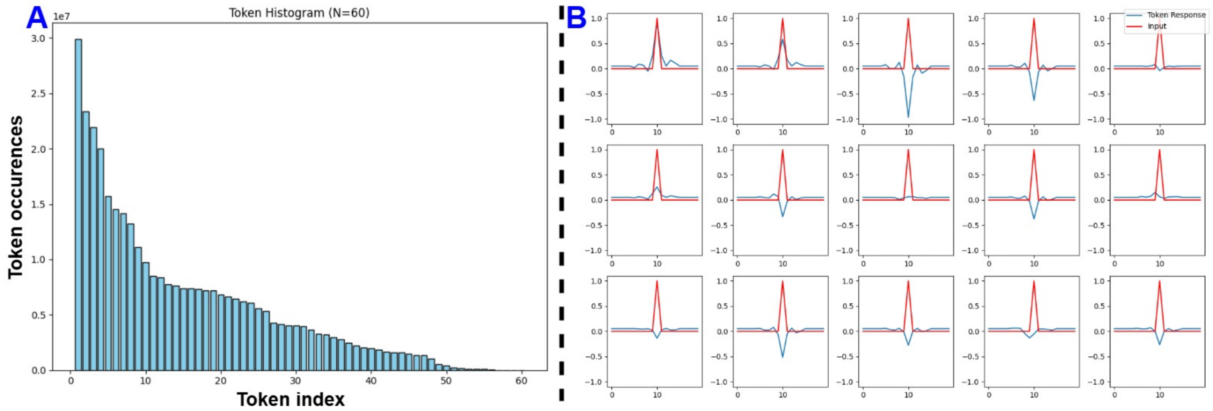

Figure A.2: **Summary plots of the learnt tokens**. (A) Histogram of token occurrences. (B) Shape of top 15 token response kernels learnt during training. Here we show the output (blue) of convolving the token kernels with a pulse (red).

B Extra details regarding MEG-GPT

B.1 Model hyper-parameters

Here we present details of the hyper-parameters used for training MEG-GPT on the Cam-CAN dataset (Table B.1) and on the Wakeman-Henson dataset (Table B.2).

**Model parameters**			
Token embedding dimension d_z	400	parcel embedding dimension d_c	400
Position embedding dimension d_p	400	Subject ID embedding dimension d_s	400
Input embedding dimension	400	Transformer model dimension d	400
Receptive field L	80	Patch size L_p	4
Number of patches P	20	Unpatched sequence length L_u	16
Latent sequence length L_{latent}	40	Number of head N_{head}	4
Number of layers N_{layer}	4	Feed forward network number of units	400
Feed forward network activation	Leaky ReLU	Feedforward network dropout	0.2
**Training parameters**			
Batch size	8	Number of epochs	60
Learning rate	1e-5	Loss sequence length L_{loss}	8

Table B.1: **Hyper-parameters for the foundation model on Cam-CAN**.

Training parameter	Value
Batch size	16
Number of epochs	10
Learning rate	5e-7

Table B.2: **Hyper-parameters for fine-tuning the foundation model on Wakeman-Henson**.

B.2 Training curves

Here we present the training curves of MEG-GPT when trained on the Cam-CAN dataset and fine-tuned on the Wakeman-Henson dataset in Figure B.1.

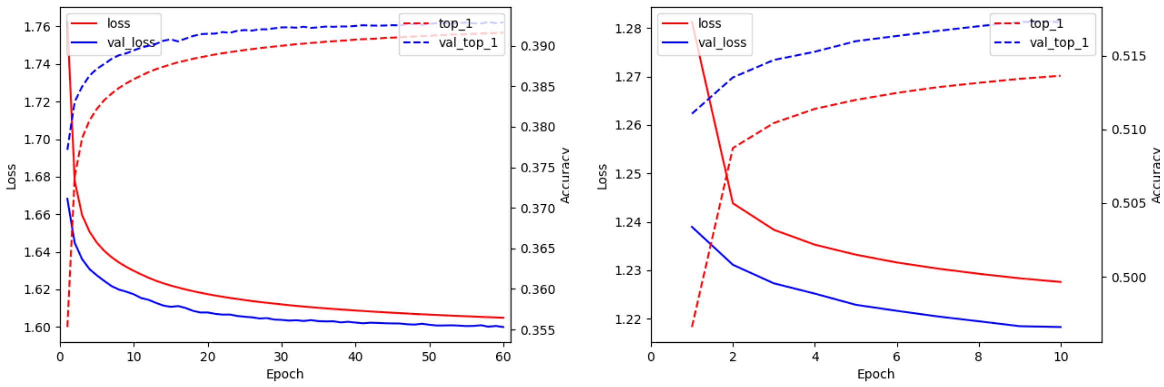

Figure B.1: **Training curves of MEG-GPT**. Left: Training curve on the Cam-CAN dataset. Right: Training curve on the Wakeman-Henson dataset.

B.3 Embedding vectors encode meaningful information through training

Here we visualise the different embedding vectors learnt during the training process, with tSNE, shown in Figure B.2. We can see that token embeddings and position embeddings are organised in their respective embedding spaces according to token frequency and position in a sequence. Furthermore, we see well-separated clusters of the parcel embeddings according to pre-defined cortical regions, and regions that are geographically close are also close in the parcel embedding space (e.g. Visual parcels are close to temporal and parietal parcels). This shows different sources of meaningful variations in the data are captured by MEG-GPT during the training process.

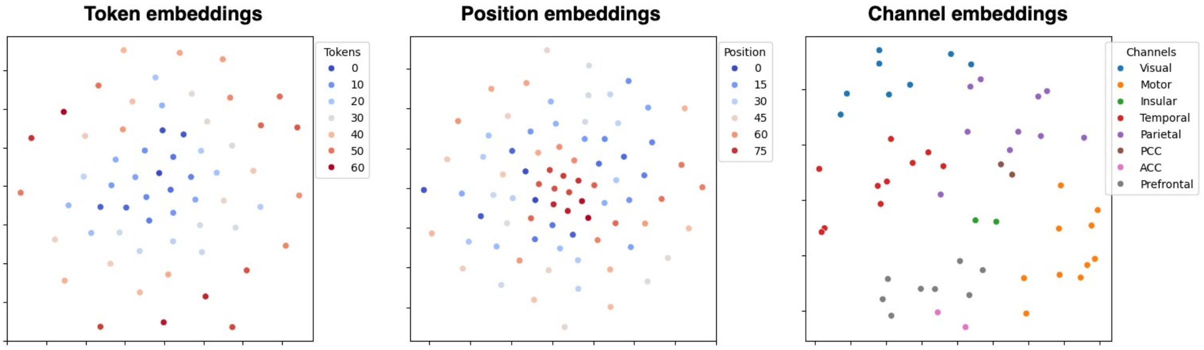

Figure B.2: **Embedding vectors encode meaningful information**. Each of the embeddings are projected with tSNE to 2 components.

C Extra details regarding subject variability

C.1 Subject-specific features

Here we describe four different ways to extract subject-specific features.

Spatial feature: This feature is designed to only include spatial information and no spectral information. For MEG data of each subject, we first calculate the PSD of the data and take the average of the PSD over frequencies for each parcel.

Spectral feature: This feature is designed to only include spectral information and no spatial information. For MEG data of each subject, we first calculate the PSD of the data and take the average of the PSD over parcels for each frequency bin.

Spatial + Spectral feature: This feature is designed to include both spatial and spectral information. For MEG data of each subject, we first calculate the PSD of the data and flatten the matrix to a vector.

Time delay embedding feature: This feature is also designed to include both spatial and spectral information. It performs the time-delayed embedding transformation (**?**) by adding lagged versions of each parcel as extra channels of the data. The static covariance matrix of the time-delay embedded data is calculated for each subject and the upper triangular part of the covariance matrix is flattened to give the required feature.

C.2 Predicting subject labels from features

Here we describe how we predict subject label of MEG-GPT generated data based on features introduced in Appendix C.1. Conceptually, for each subject i we check if feature of subject i in the generated data is closest to the feature of subject i in the real data compared to that of all other real data subjects. More formally, let x and $\hat{x}$ be the real and generated data. We further let f and $\hat{f}$ be the extracted features (any of the features introduced in Appendix C.1) from x and $\hat{x}$, respectively. Then we use a nearest neighbour classifier with correlation distance as the distance metric. More specifically, we construct the pairwise correlation distance matrix $\Sigma_{x,\tilde{x}} \in \mathbb{R}^{N_{subject} \times N_{subject}}$ such that the i, j-th entry of Σ is the correlation distance ($1-$ correlation) between subject i in the real data and subject j in the generated data. To calculate the top k accuracy, we check for each column of $\Sigma_{x,\tilde{x}}$ if the diagonal element is among the smallest k elements in the column.

C.3 Consistency score

Consistency score is a measure of the similarity between the pairwise structure in real data and generated data. We start with computing correlation matrices $\Sigma_{x,x}$ and $\Sigma_{\tilde{x},\tilde{x}}$, which are pairwise correlation matrices of the features between real data subjects and between generated data subjects respectively. Then the consistency score is defined as the correlation between the upper triangular elements of $\Sigma_{x,x}$ and $\Sigma_{\tilde{x},\tilde{x}}$.

Given the null distribution that generated data of different subjects is the same, the features extracted have the same distribution and is exchangeable. Hence we can permute the rows and columns of $\Sigma_{\tilde{x},\tilde{x}}$ and get the null distribution of the consistency score.

D Linear autoregressive model

In this paper we also trained linear autoregressive models as a baseline comparison with MEG-GPT. To account for the fact that different parcels might have different data distributions, we train independent autoregressive models on data of each of the parcels. The linear autoregressive model used in this paper has an order of 80 in order to match the receptive field of MEG-GPT.

During data generation of each parcel, an initial prompt is generated from a standard normal distribution. Then data are generated in an autoregressive manner where at each time point, Gaussian noise with standard deviation being the standard error of the regression is used.

E Hidden Markov Model for burst detection

E.1 Extra results of TDE-HMM on real data

Here we show extra results of single parcel burst detection on real data. We can see from Figure E.1B that state 3 is correlated with increased α and β power and state 2 is correlated with increase δ/θ power. In addition, we present the state covariance matrices and inferred state time courses in Figure E.1A and E.1C.

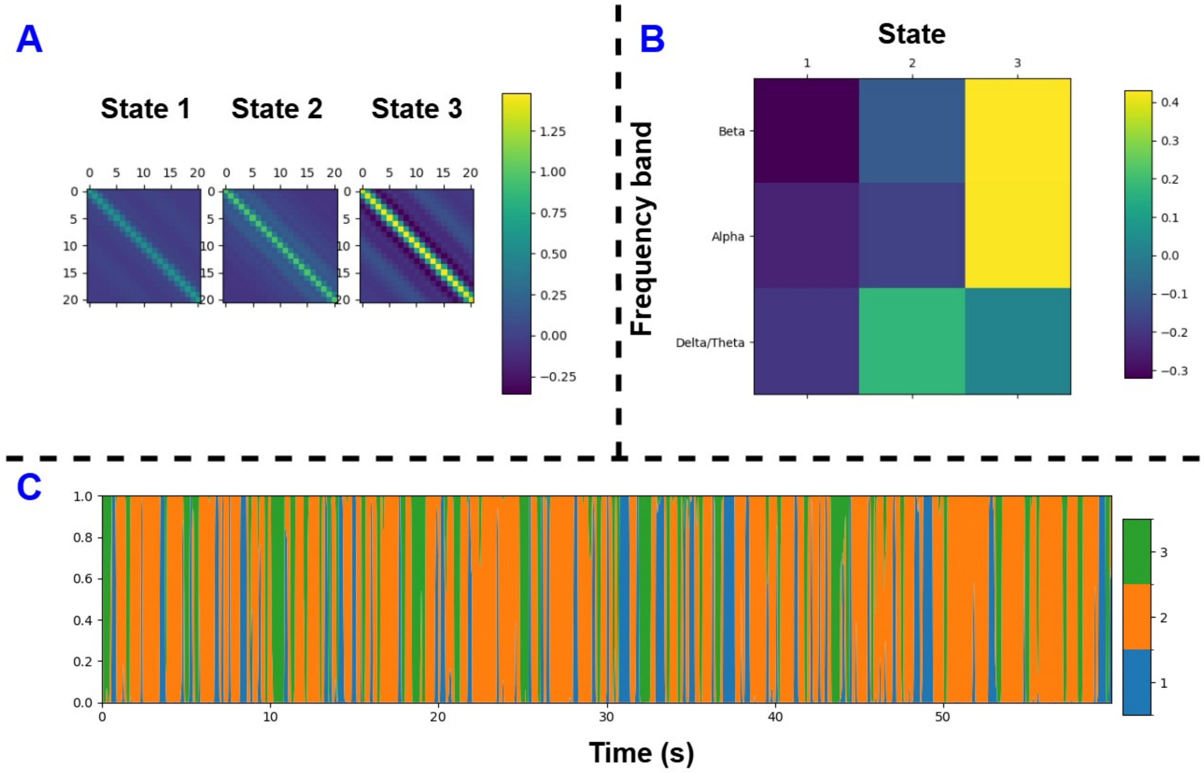

Figure E.1: **Results of TDE-HMM applied to real MEG data**. (A) Inferred state covariance matrices for the three hidden states. (B) Correlation between state time courses and power in different frequency bands. (C) Exmaple state time courses over the first 60 seconds from the first subject in the Cam-CAN dataset.

E.2 Summary statistics of HMM states

3 summary statistics of the state time courses are used in this paper. These are

- **Bursting count**: Also referred to as the switching rate, it is defined as the mean number of activations per second for each subject.
- **Mean interval**: It is defined as the mean time between state activations for each subject.
- **Mean lifetime**: It is defined as the mean time between entering and leaving a state for each subject.

E.3 Results on a visual parcel

Here we show that the results in Section 3.4 can be reproduced in another parcel. Here we choose a visual parcel, location shown in Figure E.2A.

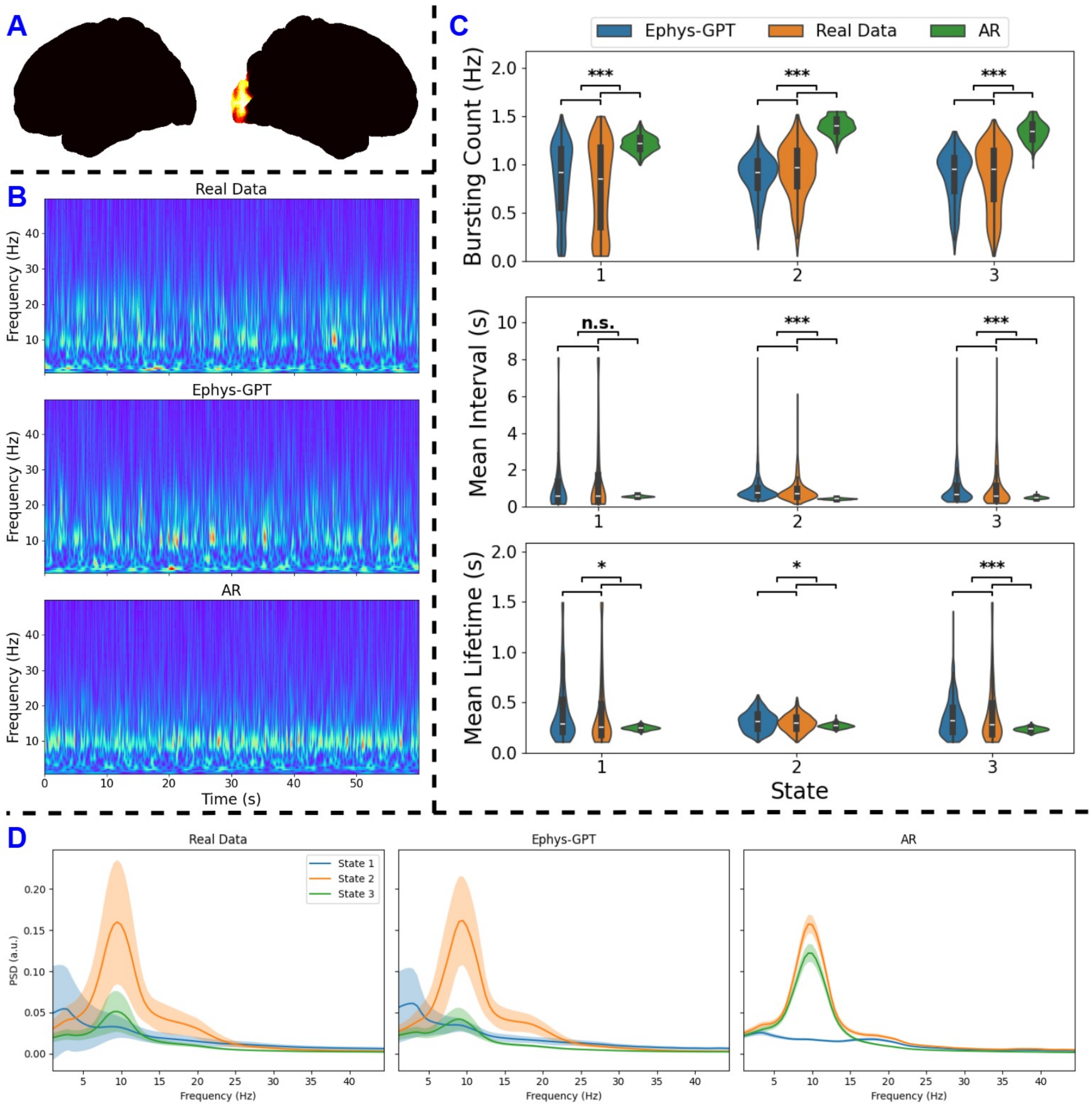

Figure E.2: **Burst detection in visual cortex**. (A) Location of the visual parcel selected. (B) Wavelet transform of the first 60 seconds from the first subject: real data (top), MEG-GPT generated data (middle), and linear autoregressive (AR) model generated data (bottom). (C) Summary statistics for each HMM state, including bursting count (top), mean interval (middle), and mean lifetime (bottom). Results for MEG-GPT are shown in blue, real data in orange, and AR model in green. Asterisks mark statistics and states where MEG-GPT results more closely match real data compared to the AR model. The asterisks (*) and (***) indicate a p-value < 0.05 and < 0.001, respectively, and "n.s." indicates a non-significant result. (D) State-specific power spectral density (PSD) profiles with the solid line representing the group average and the shaded area indicating one standard deviation across subjects.

F Extra details regarding the task decoding

F.1 Classifier

The pipelines for classification for raw epoch features and MEG-GPT extracted features are the same. Each feature is standardised with the mean and standard deviation computed using the trials in the training set. Then a multinomial logistic regression with default settings in scikit-learn v1.7.0 is used.

F.2 Other subjects as testing subject

In the main text, we have used subject 19 in the Wakeman-Henson dataset to test for out of subject accuracy. Here we repeated the analysis for the baseline and the zero-shot features using other subjects to test for out of subject accuracy. Fine tuning was omitted because it was not computationally feasible to do the fine-tuning training in each iteration of the cross-validation loop. The results are shown in Figure F.1, and we see that the improvements in both within subject and out of subject accuracy are consistent over the different testing subjects.

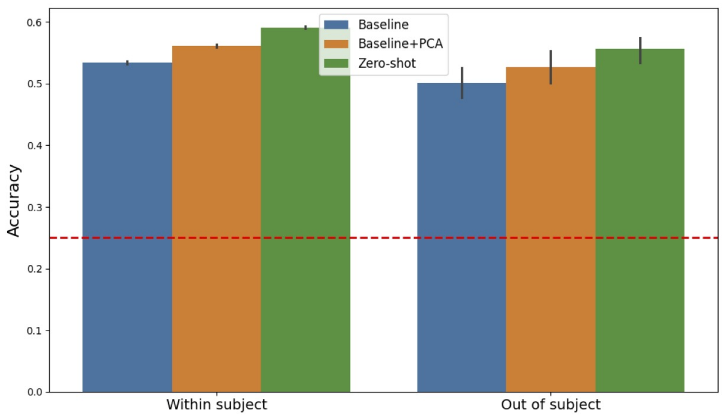

Figure F.1: Decoding accuracy across all subjects. Here the error bars are 95% confidence interval across subjects.

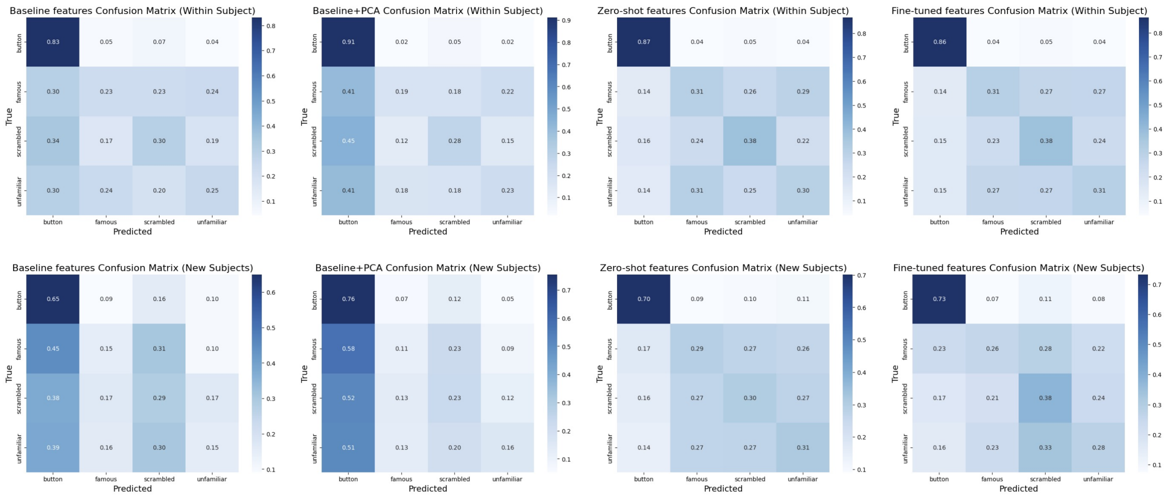

Figure F.2: Confusion matrices of Within Subject prediction (top row) and for New Subject prediction (bottom row) are plotted for baseline features, baseline+PCA features, zero-shot features, and fine-tuned features.

G Simulation ablation study

To investigate the role of embedding components of `MEG-GPT`, we performed a simple controlled simulation study. We simulate the data with 12 parcels for 60 subjects where

- Each subject's data is 5 minutes long at a sampling frequency of 100Hz.
- To introduce channel differences, parcels 1-4 has base bursting frequency at 3Hz, parcels 5-8 at 10Hz, and parcels 9-12 at 17Hz.
- To introduce subject differences, we applied a -0.5Hz frequency shift to all channels of the first 30 subjects and +0.5Hz for the last 30 subjects.

This results in a structured dataset where parcel-wise structure is defined by the base frequency groups and subject-level structure is defined by a global frequency shift.

We trained 3 variants of `MEG-GPT` on this simulated dataset:

- Full model: includes both parcel and subject embeddings.
- Without subject embedding: removes subject-level information.
- Without parcel embedding: removes parcel-specific information.

Then we generated data and computed the PSD for each channel. In Figure G.1, each row corresponds to a channel and the two curves represent two subject groups. We can see that the full model successfully captures both the channel-specific frequency structure and the subject-level frequency shifts. The PSDs clearly separate the two subject groups while preserving the correct base frequencies across channels. For model without subject embedding, the generated data captures the channel-specific frequencies but fails to distinguish between subject groups. The PSDs for the two groups largely overlap, indicating that subject-level variability is not learned. For the model without parcel embedding, the model fails to preserve the channel-specific frequency structure and the PSDs became all mixed up, i.e. the spatial (parcel-wise) organisation is not properly captured.

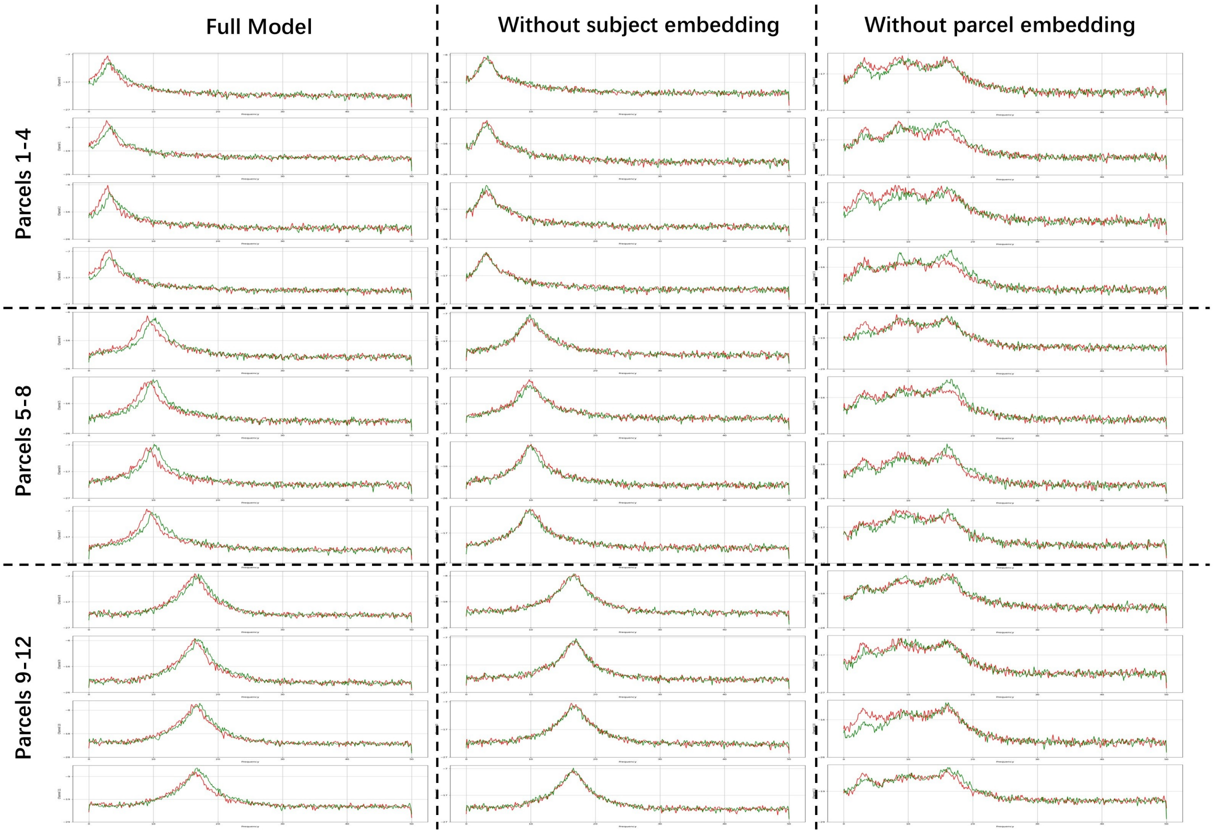

Figure G.1: **Ablation study on embedding components using simulated data**. Each row corresponds to a channel. Red and green curves represent the PSDs of the two subject groups. Left: full model. Middle: model without subject embeddings. Right: model without channel embeddings. The full model captures both channel-specific frequencies and subject-level shifts, while removing either embedding degrades performance in a targeted way.
